# Regulation of *hedgehog* Ligand Expression by the N-End Rule Ubiquitin-Protein Ligase Hyperplastic Discs and the *Drosophila* GSK3β Homologue, Shaggy

**DOI:** 10.1371/journal.pone.0136760

**Published:** 2015-09-03

**Authors:** Sophie Moncrieff, Matthieu Moncan, Flavia Scialpi, Mark Ditzel

**Affiliations:** MRC Institute of Genetics and Molecular Medicine at the University of Edinburgh, Edinburgh CRUK Cancer Research Centre, Western General Hospital, Crewe Road South, Edinburgh, EH4 2XR, United Kingdom; University of Massachusetts Medical School, UNITED STATES

## Abstract

Hedgehog (Hh) morphogen signalling plays an essential role in tissue development and homeostasis. While much is known about the Hh signal transduction pathway, far less is known about the molecules that regulate the expression of the *hedgehog (hh)* ligand itself. Here we reveal that Shaggy (Sgg), the *Drosophila melanogaster* orthologue of GSK3β, and the N-end Rule Ubiquitin-protein ligase Hyperplastic Discs (Hyd) act together to co-ordinate Hedgehog signalling through regulating *hh* ligand expression and Cubitus interruptus (Ci) expression. Increased *hh* and Ci expression within *hyd* mutant clones was effectively suppressed by *sgg* RNAi, placing *sgg* downstream of *hyd*. Functionally, *sgg* RNAi also rescued the adult *hyd* mutant head phenotype. Consistent with the genetic interactions, we found Hyd to physically interact with Sgg and Ci. Taken together we propose that Hyd and Sgg function to co-ordinate *hh* ligand and Ci expression, which in turn influences important developmental signalling pathways during imaginal disc development. These findings are important as tight temporal/spatial regulation of *hh* ligand expression underlies its important roles in animal development and tissue homeostasis. When deregulated, *hh* ligand family misexpression underlies numerous human diseases (e.g., colorectal, lung, pancreatic and haematological cancers) and developmental defects (e.g., cyclopia and polydactyly). In summary, our *Drosophila*-based findings highlight an apical role for Hyd and Sgg in initiating Hedgehog signalling, which could also be evolutionarily conserved in mammals.

## Introduction

Hh morphogens act in multicellular animals to control development and homeostasis of adult tissues and organs [1, 2]. In *Drosophila*, the Hh pathway (HhP) governs many aspects of *Drosophila* development that includes adult eye and head development from the larval eye-antennal imaginal disc (EA disc)[3]. In an unstimulated cell, the unbound Hh-receptor Patched (Ptc) constitutively represses Hh signalling by indirectly suppressing the pathway’s transcriptional effector Cubitus Interruptus (Ci). Whereupon Hh ligand stimulation, Ci activity is de-repressed to permit of expression of Ci’s target genes[2].

The phosphorylation-directed threonine/serine kinase Sgg plays as important role in suppressing Ci activity, as well as being implicated in a diverse array of signal transduction pathways that include insulin, stress, growth factor, cytokine and morphogen signalling[4]. Within the HhP, Sgg, together with Protein kinase A and Casein Kinase I[5, 6], phosphorylate Ci to create a binding site for the F-box protein Slimb (Slmb, the Drosophila homologue of mammalian βTrCP)[7]. This phosphodependent interaction allows the Slmb-bearing Cullin-1 E3 complex (Cul1^Slmb^) to promote Ci ubiquitylation and its subsequent partial proteolysis. Removal of Ci’s C-terminal transcriptional transactivation domain converts full-length 155kDa Ci (Ci^155^) into a 75kDa Ci (Ci^75^) transcriptional repressor. As part of a negative feedback mechanism, an alternative Cullin-3 based complex (Cul3^Rdx^) also targets Ci^155^ for ubiquitin-dependent proteasomal degradation using the substrate specificity factor, Roadkill (Rdx), the *Drosophila* homologue of Speckle-type POZ protein (SPOP)[8, 9].

Although much is known about the molecular mechanisms governing Ci^155^ expression in the Hh-stimulated cell, far less is know about the upstream events that govern the expression of the *hedgehog* ligand. Hyperplastic Discs (Hyd), a ubiquitin-protein ligase (E3) of the N-end rule pathway[10] represents one of the few non-transcription factors identified as a suppressor of *hh* ligand expression. Hyd was originally identified as a regulator of imaginal disc development, with *hyd* mutant alleles resulting in either hyperplastic or hypoplastic discs[11]. Hyd contains a number of domains related to ubiquitin signalling, which include a ubiquitin binding domain[12], a substrate recruitment domain for N-end rule substrates[13] and a catalytic HECT domain[14]—the presence of which defines Hyd as an E3 ubiquitin-protein ligase. While little is known about Hyd’s molecular functions outside of the HhP, its mammalian orthologues are implicated in DNA damage signalling[15–17], miRNA activity[18], metabolism[19] and cell cycle checkpoint control [20–23].

Previous work by Lee et al[24] revealed that *hyd* mutant (*hyd* ^*K3*.*5*^) clone-bearing EA discs were hyperplastic, spatially misexpressed *hh* and exhibited increased Ci^155^ levels within clones[24]. Deletion of *hh* function within the *hyd* ^*K3*.*5*^ mutant clones partially rescued the EA disc overgrowth phenotype, but did not rescue the increased levels of Ci^155^ expression. Therefore suggesting that Hyd can normally suppresses Ci^155^ expression independent of any effect mediated by *hh* ligand overexpression. These results indicated that Hyd may have independent roles in controlling the (i) initiation of Hh signalling by regulating *hh* ligand expression and (ii) modulating the pathway response by governing Ci^155^ expression. What remained unclear from this elegant work was the underlying molecular mechanism by which Hyd might independently regulate *hh* and Ci^155^ expression?

Here we identify a genetic interaction between *hyd* and *sgg* in the regulating HhP activity in the developing EA disc. Our work reveals a previously unreported role for Sgg in regulating *hh* ligand expression, while identification of a physical interaction between Hyd, Sgg and Ci^155^ provides a potential mechanism by which Hyd could influence both *hh* ligand and Ci^155^ expression patterns. Overall, these findings provide new mechanistic insights into how Hyd and Sgg influence different aspects of Hh signalling.

## Materials and Methods

### Plasmids

Constructs were made by standard PCR-based cloning methods using restriction enzyme cloning. *hyd* and *EDD* inserts were ligated into a modified *pMT* or *pcDNA5* vector (Invitrogen) containing an N-terminal HA-Strep tag. *hyd* mutant constructs were constructed using standard site-directed mutagenesis using primers targeting: UBR^mt^ (C1272A+ C1274A), PABC^mt^ (Y2509A+C2527A) and HECT^mt^ (C2854A) domains. Inserts for *sgg* were cloned into a C-terminal V5/FLAG-tagged *pMT* or *pcDNA5* vector. *Myc-GLI2* expression vector was kindly provided by Rune Toftgard (Karolinska Institute, Sweden). *Drosophila* cDNAs were acquired from the *Drosophila* Genomics Resource Centre, DGRC. *UAS-hyd* ^*WT*^ and *hyd* ^*C>A*^ (HECT^mt^ C2854A) inserts (NotI-NotI) were cloned into *pUAST* and sent to Bestgene Inc., USA for transgenic production.

### Genomic DNA sequencing

*hyd* alleles were sequenced by Sanger sequencing using an array of overlapping primers. Sequences were aligned against *hyd* genomic DNA using SerialCloner software.

5’ FL hyd ATG: GTTTCCATGCAATTTGTTTTGCAACC

5' hyd genomic @2580: CGAAAGAAGCTTGCAGAAGTCCATGC

5' hyd genomic @3104: CTTGACTTGACCAAATCAGACGC

5' hyd genomic @3627: CGTGCCCGAAGACCTTATCTCCCTGCTGG

5' hyd genomic @4156: GGATATCTGAAGAATTGCAGC

5' hyd genomic @4680: CGCCGCTTCTTGTGGGACAAATTCCGG

5' hyd genomic @5211: GTGAAGGACGTGGTGTTTGTCG

5' hyd genomic @5736: GTGCTTCGTGATGGCAATGGAGC

5' hyd genomic @6255: GCAACTATGAGTTCATCCGCTGCCGG

5' hyd genomic @6779: GCTAAAGGAGGCCATGATTTTCCCG

5' hyd genomic @7302: GATAATGATATGCCGGACCATGATCTGGAGC

3’seq hyd@4536: AACACAGCTCTGCACGTATTTGTTGC

5'hyd@3000: CTCGACAAGCGCTTACGTTAG

5' hyd genomic @6779: GCTAAAGGAGGCCATGATTTTCCCG

5' hyd genomic @7302: GATAATGATATGCCGGACCATGATCTGGAGC

5' hyd genomic @7775: GCTGCACAAGATATCCATCGAGG

5' hyd genomic @8309: GGACGGCATGCAAGATGACGAGAGC

5' hyd genomic @8839: CGACAACGGCCAGCAACTTGGC

5' hyd genomic @9384: GCTCACACACCTCTGAGCACCGAGACG

5' hyd genomic @9955: CGATTCTAGTAAGACGGGTGATGG

5' hyd genomic @10505: GCCGCTGGAAGCTAACTCTGG

5' hyd genomic @11029: CGTTCGGCCCGTGAGAGGAAGG

5' hyd genomic @11570: GCCAAGGCTTTGCATCATTCGAGCG

5' hyd genomic @12088: GGAGGTATGGGCAAATATTGCG

5' hyd genomic @12548: CGACTGCGAATACTTGTATCTCTCGG

3’ Hyd 3'UTR: TGGCCGTTTTATTGGTTACAATGG

### Cell culture

Insect S2 and Cl8 cells were acquired from, and cultured according to, the Drosophila RNAi Screening Centre (DRSC, USA). Transfections were performed using Effectene (QIAGEN) according to the manufacturer’s protocol and protein expression induced with 0.35 mM CuSO_4_. HEK293 (CRL-1573) cells were cultures according to ATCC guidelines and transfected using CaCl_2_ (Life Technologies).

### Pull-down assays, co-IP and Western blotting

Cells were processed for immunoprecipitation (IP) and/or SDS-PAGE/Western blotting as previously described in[25]. Briefly, cells were lysed 48h post-transfection Triton lysis buffer (50mM Tris pH 7.5, 100mM NaCl, 2mM EDTA, 1% Triton X-100, 1X Roche protease inhibitor mix, 1X Roche phosphatase inhibitor mix). Post clarification, HA-Strep Hyd was pulled down using either Streptactin sepharose (GE Healthcare) or HA-agarose (clone HA-7) (Sigma) for 1hr at 4°C with rotation. After washing, protein complexes were eluted with one bead volume of 1X NuPAGE LDS Sample Buffer (Invitrogen) and 100mM DTT. To IP endogenous Hyd, 5μl M19 antibody (Santa Cruz) was added to the lysate and incubated at 4°C with rotation for 2 h, followed by Protein-A agarose (Sigma) for 30 min with rotation. Samples were run on BIS-TRIS-gradient gels (Invitrogen) and blotted onto PVDF (Millipore). Antibodies used were: mouse HA (1:2,000 Covance), FLAG M2 (1:2,000 Sigma), SGG GSK-4G-AS (1:5,000 Euromedex), Myc 9B11 (1:6,000 Cell Signalling), V5 (1:2000 AbD Serotec); goat EDD M19 (1:1,000; Santa Cruz); rat Ci 2A1 (1:10 DSHB); mouse, goat and rat HRP-conjugated secondary antibodies were used 1:5,000 (Jackson ImmunoResearch Laboratories).

### Fly stocks

Alleles used are described in Flybase except the UAS-hyd lines that are described here for the first time. *hyd* ^*K7*.*19*^ and *hyd* ^*K3*.*5*^ were obtained from Jessica Treisman (NYU School of Medicine, New York, NY, USA) while the others were either created using pUAST-mediated transgenesis or purchased from the Bloomington *Drosophila* Stock Centre. *Sp//SM6-TM6* was obtained from Marcos Vidal (Beatson Institute for Cancer Research, Glasgow, UK). Flies were maintained on standard medium at 25°C.

The following lines were created and used for mitotic clone analysis:

yw ey-flp^3.6^; act>y+>GAL4, UAS-GFP; FRT^82B^, tubGAL80//SM6-TM6

FRT^82B^, hh-lacZ/TM6B Tb

FRT^82B^, hyd^K7.19^ hh-lacZ/TM6B Tb

UAS-sgg^S9A^; FRT^82B^, hyd^K7.19^ hh-lacZ//SM6-TM6 Tb

UAS-sgg^S9A^; FRT^82B^, hh-lacZ//SM6-TM6 Tb

UA-sgg^-RNAi^; FRT^82B^, hyd^K7.19^ hh-lacZ//SM6-TM6 Tb

UAS-sgg^-RNAi^; FRT^82B^, hh-lacZ//SM6-TM6 Tb

UAS-hyd^WT^ or-hyd^C>A^; FRT^82B^,/TM6B Tb

UAS-hyd^WT^ or-hyd^C>A^; FRT^82B^, hyd^K7.19^ /TM6B Tb

The following lines were created and used for wing analysis:

Vg-GAL4

Vg-GAL4; UAS Sgg^S9A^ / +

Vg-GAL4; UAS Sgg^S9A^ / UAS hyd^WT^

Vg-GAL4; UAS Sgg^S9A^ / UAS hyd^C>A^

Vg-GAL4; UAS Sgg^RNAi^ / +

Vg-GAL4; UAS Sgg^RNAi^ / UAS hyd^WT^

Vg-GAL4; UAS Sgg^RNAi^ / UAS hyd^C>A^

### Fly Crosses and Clone Production

#### Eye disc

Using a MARCM-based approach[26] GFP-labelled mitotic clones were generated using *ey-flp* to recombine *FRT82B* sites and remove a STOP cassette preventing expression of *act-GAL4* to drive *UAS*-response elements (*UAS-GFP* and-*cDNAs* and-*RNAi*). Use of *FRT82B tub-GAL80* ensured expression of *UAS*-response elements were tightly regulated. For *hh* expression studies *hh-lacZ* ^*P30*^[27] was recombined onto *FRT82B* and *FRT82B hyd* ^*k7*.*19*^. Three-hour embryo collection windows were used to synchronise L3 collection for dissection of imaginal eye discs.

#### Wing disc

*UAS*-*sgg* and *hyd* overexpression in the wing disc was mediated by *vg*-*GAL4* or *sca*-*GAL4* expression. Adult wings were imaged 16hrs after emerging from pupae.

### Immunofluorescence

L3 eye-antennal discs were dissected in PBS as previously described[28]. Discs were incubated in primary antibodies overnight at 4°C and incubated with secondary antibodies for 2 hours at room temperature, followed by washing and mounting in Vectashield containing DAPI (Vector Laboratories, Inc.). All antibodies were diluted in blocking solution. Primary antibodies used were mouse β-Gal (1:100; Developmental Studies Hybridoma Bank, DSHB), Ptc (1:10; DSHB); rabbit Hh (1:400)[29]; rat Ci 2A1 (1:10; DSHB). Secondary antibodies were mouse, rabbit and rat conjugated to Alexa 594 and Cy5 (1:500; Invitrogen).

### Image acquisition and data analysis

Confocal images were captured on a NikonA1R confocal microscope at 20X or 60X magnification. Widefield image sections were captured at 20X on a Zeiss Axioplan II and images deconvolved using Volocity (PerkinElmer). For quantitative analysis, imaged were taken using the same acquisition parameters. Brightfield colour images of heads, wings and notum were acquired using an Olympus SX9 stereomicroscope (4X) attached to a Nikon D300 camera. Image J was used to create a black overlay mask by thresholding the GFP channel images. The subsequent black mask corresponding to GFP negative regions was then superimposed over the Ci^155^ or βGal images. ImageJ was also used for measuring adult heads, band densitometry and pixel intensity. Microsoft Excel and GraphPad Prism were used for graphs and ANOVA and t-test statistical analysis.

## Results

### Hyd binds Ci^155^ and the Drosophila GSK3β homologue Shaggy

**Fig 1:**
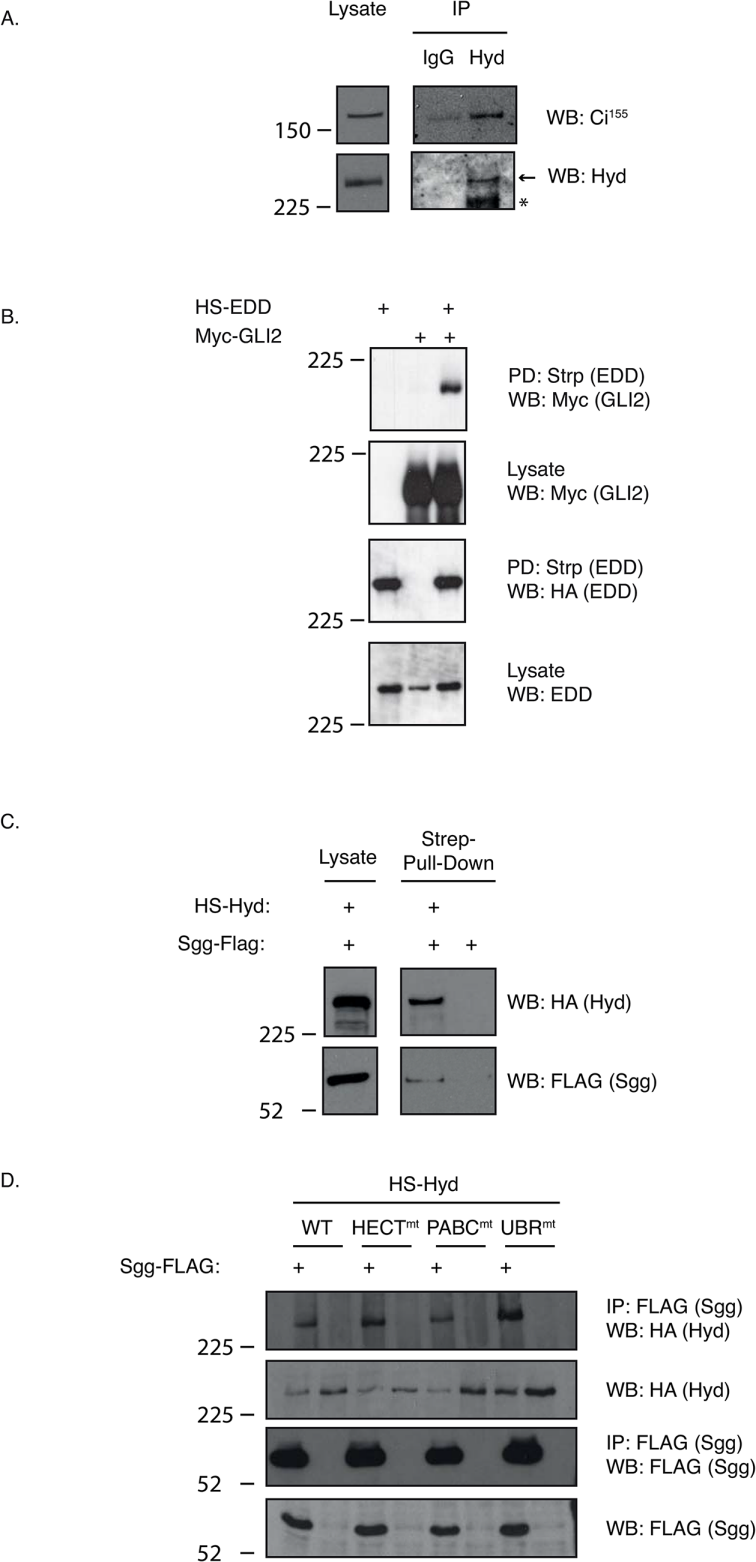
Hyd binds the Hedgehog pathway’s key transcriptional effector Ci^155^ and the Ci-regulatory kinase Sgg. Co-immunoprecipitation (A,D) and affinity-purification (B,C) studies with the indicated affinity reagents were examined by SDS-PAGE and Western blotting with the indicated antibodies. (A) *Drosophila* CL8 cells were lysed and incubated with either Hyd or control IgG antibodies and affinity purified by Protein G beads. An arrow indicates the position of the expected size band and an asterisk indicates the presence of an uncharacterised faster migrating Hyd species. (B) Mammalian HEK293 cells were transfected with the indicated constructs and lysates underwent Streptactin-mediated purification (Strp) to purify Haemagglutinin-Streptactin-EDD (HS-EDD) and detect co-purified Myc-GLI2. (C) *Drosophila* S2 cells were transfected with either *HS-hyd or HS-vector* control, lysed and then incubated with Streptactin-affinity resin. Control and Hyd-coated beads were then incubated with Sgg-FLAG expressing S2 lysate and, following washing, analysed for bound Sgg-FLAG. Only the HS-Hyd beads purified FLAG-Sgg. (D) *Drosophila* S2 cells were co-transfected with the indicated *hyd* mutant and *sgg*-*FLAG* constructs and FLAG-affinity purified complexes were analysed with the indicated antibodies.

We initially sought to gain a molecular insight into how Hyd might directly regulate Ci^155^ expression by addressing whether Hyd could physically interact with Ci^155^. *Drosophila* wing-disc-derived CL8 cells that express both Hyd and Ci^155^ were used to investigate endogenous protein interactions. Co-immunoprecipitations (Co-IPs) revealed that Hyd consistently co-purified endogenous Ci^155^ at levels significantly increased over IgG control levels (Fig 1A). To support these observations we addressed whether Hyd’s human homologue, EDD, could also bind one of the human Ci homologues, GLI2. Exogenously expressed Haemagglutinin-Streptavidin-(HS)-EDD efficiently co-purified full-length Myc-tagged GLI2 from transfected HEK293 cells (Fig 1B). In summary, our data indicated that both Hyd and EDD bind to the HhP's major transcriptional effectors.

A recent report revealed EDD’s interaction with GSK3β[30], a known GLI2 binding protein[5, 6]. This observation provided a potential means of Hyd to indirectly interacting with Ci and prompted us to examine if Hyd could also co-purify GSK3β’s *Drosophila* homologue, Shaggy (Sgg) (Fig 1C and 1D). HS-Hyd was first purified from *HS-hyd* transfected S2 cells using Streptactin-affinity resin. Control or HS-Hyd-loaded beads were then incubated with Sgg-FLAG expressing S2 cells lysate. HS-Hyd bound resin, but not control resin, co-purified Sgg-FLAG (Fig 1C). To identify which domains might be important for mediating/promoting the interaction with Sgg, we created a series Hyd constructs with predicted loss-of-function point mutations: HECT(C2854A)[14] to potentially improve the interaction by preventing HECT-mediated ubiquitylation and degradation; and PABC(Y2509A+C2527A)[31] and UBR(C1272A + C1274A)[13] domains to unfold these protein-protein interaction domains. None of the mutants altered the amount of co-purified FLAG-Sgg (Fig 1D), suggesting that other domains/residues are important for Hyd’s interaction with Sgg. Taken together this evidence reveals an evolutionarily conserved ability of Hyd and EDD to bind both the HhP’s key transcriptional effector (Ci/GLI2) as well as one of its key regulatory kinases (Sgg/GSK3β).

### The *hyd* ^*K7*.*19*^ allele lacks E3 function and promotes adult head defects

**Fig 2:**
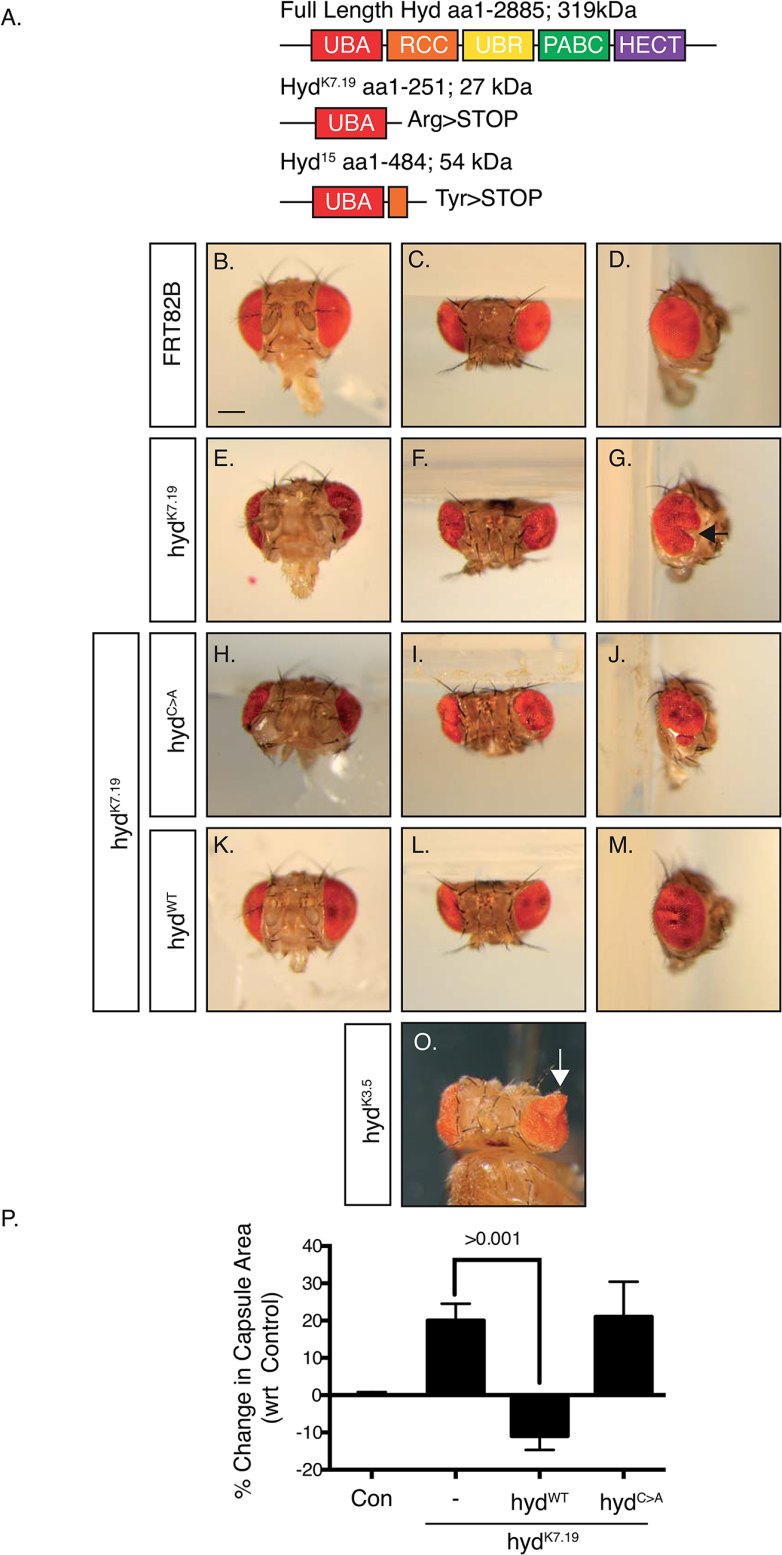
*hyd* ^*K7*.*19*^ is defective in HECT E3 function and causes abnormal head development. (A) Schematic representation of the full length Hyd protein containing the Ubiquitin Association Domain (UBA), Regulator of Chromatin Condensation-like (RCC), Ubiquitin-Protein Ligase E3 Component N-Recognin (UBR) domain, Poly(A)-Binding Protein C-Terminal (PABC) and Homologous to the E6AP Carboxyl Terminus (HECT) domains and the potential protein products encoded by *hyd* ^*k7*.*19*^ and *hyd* ^*15*^. In comparison to control heads (B-D), *hyd* ^*k7*.*19*^ flies (E-G) exhibited disruption of the adult eye and increased head-capsule area. Co-expression of the *hyd* ^*WT*^ (K-M), but not *hyd* ^*C>A*^ (H-J), transgene suppressed the *hyd* ^*k7*.*19*^ phenotype. Scale bar = 200μm. (O) *hyd* ^*K3*.*5*^ flies exhibit eye tissue outgrowths that are not present in *hyd* ^*k7*.*19*^ heads. (P) Quantification of the head capsule area of the indicated genotype. % values are normalised to control. n = >10 of each genotype. s.e.m and indicated p value determined by Student’s t-test.

Due to Sgg’s physical interaction with Hyd we wished to determine if perturbed Sgg function would alter the *hyd* mutant phenotype. As distinct *hyd* alleles mediate dramatically different effects on imaginal discs[11], we wished to first molecularly characterise a selection of *hyd* loss of function mutant alleles. Four available *hyd* alleles (*hyd* ^*15*^, *hyd* ^*K3*.*5*^, *hyd* ^*K7*.*19*^ and *hyd* ^*wc461*^) were sequenced to identify nucleotide changes. Analysis revealed nonsense mutations in *hyd* ^K7.19^ (aaR251>STOP) and *hyd* ^*15*^ (aa485W>STOP) (Fig 2A), but failed to find exon- or intron-associated mutations in *hyd* ^*K3*.*5*^ or *hyd* ^*wc461*^. The lack of exon/intron-associated mutations in *hyd* ^*K3*.*5*^ and *hyd* ^*wc461*^ suggested these harboured mutations in regulatory regions governing *hyd* mRNA expression, stability or translation. We chose to carry out all our studies using the most severe truncating mutation *hyd* ^*K7*.*19*^ that, if expressed, would lack all domains apart from the Hyd’s N-terminal UBA domain. Such a protein would therefore lack its ability to bind N-end rule substrates (via it UBR domain)[13], influence miRNA function (via its PABC domain)[18] and function as an E3 enzyme (via its HECT domain)[14] (Fig 2A).

To generate *hyd* ^*K7*.*19*^ clones throughout the developing EA disc we utilized mitotic recombination—a technique that permits creation of homozygous mutant cells from heterozygous tissue. The MARCM-based system[26] was used with an *eyeless* promoter driven Flippase (FLP) in combination with a FLP Recombination Target (FRT) marked *hyd* ^*K7*.*19*^ *-*bearing chromosome (FRT82B *hyd* ^*K7*.*19*^, herein referred to as simply hyd^K7.19^). This then allowed us to create homozygous *hyd* ^*K7*.*19*^ mutant clones specifically within the developing EA disc. Homozygous mitotic clones were also positively marked by GFP expression through FLP-mediated removal of an *FRT*-STOP-*FRT* signal upstream of UAS-GFP transgene. The presence of *tub*-*GAL80* on the *FRT82B* chromosome also ensured that GAL4-mediated transcription only occurred in FRT82B *hyd* ^*k7*.*19*^ homozygous cells.

Animals bearing *hyd* ^*K7*.*19*^ clones in the EA discs were viable, but exhibited dramatic changes in the shape and size of the adult eye together with a significant expansion of the head capsule (Fig 2, compare FRT82B control B-D with E-G). Over 90% of all *hyd* ^*k7*.*19*^ flies showed a ‘puckered’ eye phenotype, reflecting ingress of head capsule at the expense of the eye field along the dorsal-ventral (DV) midline (Fig 2G, arrow). Interestingly, while *hyd* ^*k3*.*5*^ adult heads showed the same eye and heads defects they also exhibited outgrowths from the eye (Fig 2O, arrow)[24] that were never observed in *hyd* ^*K7*.*19*^ heads. Such phenotypic variations potentially reflected the distinct molecular defects associated with the different alleles—an effect commonly observed across allelic series.

To confirm that the *hyd* ^*K7*.*19*^ phenotype was solely due to perturbed *hyd* function we attempted to rescue the mutant phenotype through expression of wild-type *hyd* transgene. Expression of a wild type *UAS-hyd* (*hyd* ^*WT*^) (Fig 2H–2J), but not an E3-catalytic dead *hyd* mutant (*hyd* ^*C>A*^) (Fig 2K–2M), transgene rescued the *hyd* ^*K7*.*19*^ phenotype. Quantification of the area of head capsule revealed a significant reduction in *hyd* ^*K7*.*19*^ flies expressing the *hyd* ^*WT*^, but not *hyd* ^*C>A*^, transgenes (Fig 2P). These results indicated that the gene mutation(s) associated with the *hyd* ^*K7*.*19*^ allele were effectively suppressed by the *hyd* ^*wt*^, but not the *hyd* ^*C>A*^, transgene. Therefore, it is most likely that the *hyd* ^*k7*.*19*^ phenotype is associated with loss of Hyd’s E3 catalytic activity.

### Loss of *hyd* function increases Ci^155^ and Ptc expression

**Fig 3:**
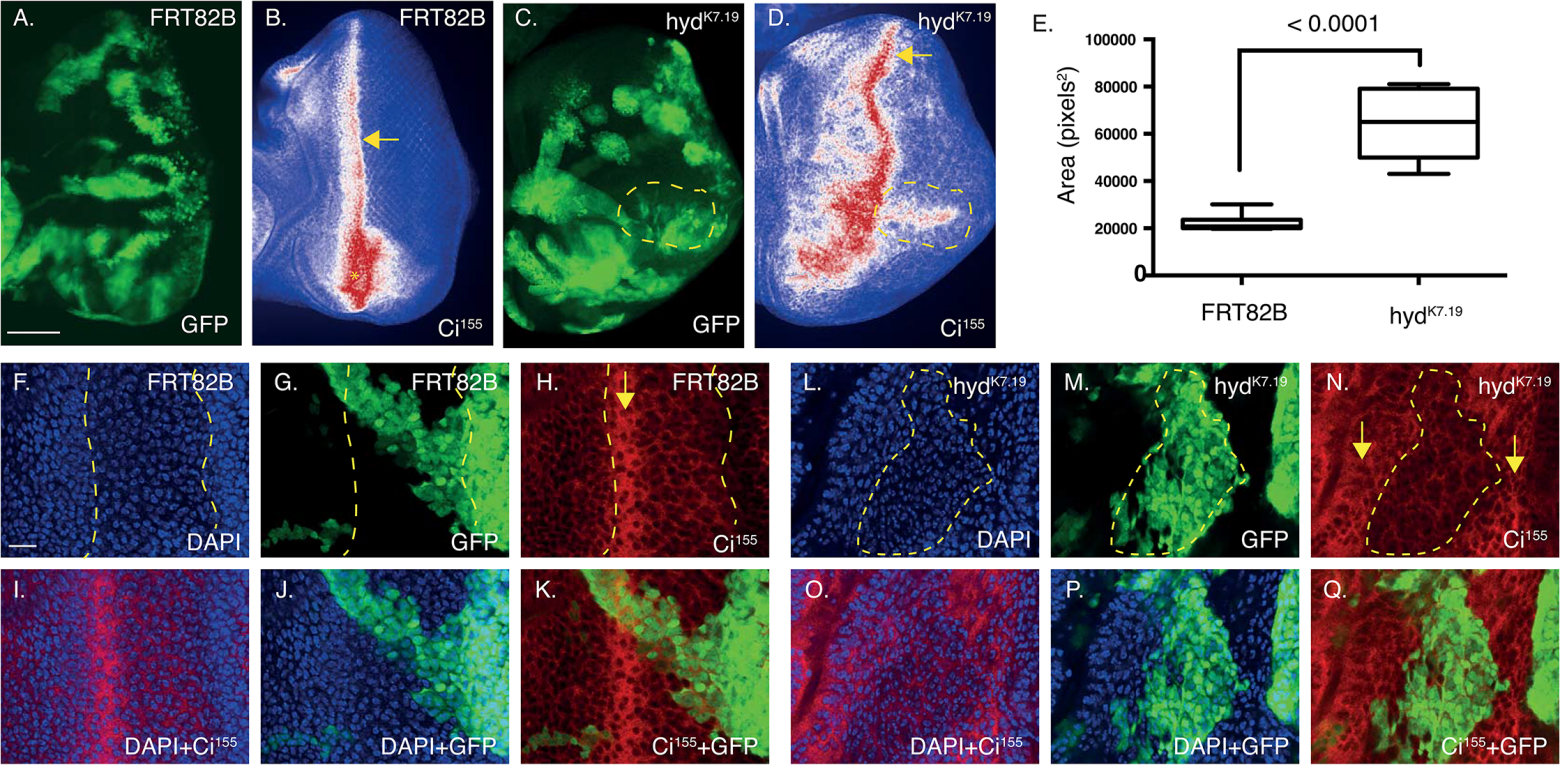
*hyd* ^*K7*.*19*^ EA discs exhibit aberrant Ci^155^ expression patterns and morphogenetic furrow-associated features. (A-D) Deconvolved widefield and confocal image (F-Q) sections of control *FRT82B* (A,B, F-K)) and *hyd* ^*k7*.*19*^ (C,D, L-Q) EA discs imaged for direct GFP fluorescence (A,C,G,K), Ci^155^ immunofluorescence (B,D,H,I,K) and DAPI (A,B,C,E,F,G). (A-D) *hyd* ^*k7*.*19*^ EA discs exhibit abnormal Ci^155^ expression patterns. A “Union Jack” lookup table was applied to Ci^155^ images to visualise low (blue), medium (white) and high (red) intensity levels and arrows marks the presumed Ci^155^ DVS / morphogenetic furrow and an asterisk indicates increased Ci^155^ staining as a result of the tissue folding over in itself (B). (D) *hyd* ^*k7*.*19*^ EA discs exhibited ectopic Ci^155^ expression in the posterior compartment (E, marked by a dashed yellow line, which is also overlaid onto C). (E) Quantification of the area of medium-to-high Ci^155^ signal in control and *hyd* ^*k7*.*19*^ EA discs. n = 5, s.e.m and indicated p value determined by Student’s t-test. (F-Q) *hyd* ^*k7*.*19*^ EA discs exhibit abnormal markers of the morphogenetic furrow. Control FRT82B EA discs exhibited normal nuclei distribution (F) and DVS Ci^155^ expression (H), while *hyd* ^*k7*.*19*^ discs exhibited irregular patterns (L,N). (F-H) Dashed lines indicated the DVS’s associated high anterior and low posterior Ci^155^ expression margins (H), which is overlaid onto (F,G). (L-N) A region of low Ci^155^ expression flanked by two DVS-like regions of high Ci^155^ expression is marked by a dashed outline (N), which is overlaid onto (L,M). Arrows mark high Ci^155^ DVS (H), or DVS-like (N), signals. Scales bars (A-D) 50μm and (F-Q) 10μm.

Once we had characterised and validated the *hyd* ^*k7*.*19*^ allele we then wished to investigate Ci^155^ expression patterns. Based on Hyd’s physical interaction with Sgg and Ci^155^ we predicted that Ci^155^ expression patterns would be altered. GFP positive mitotic clones in 3^rd^ instar larval EA discs (Fig 3A and 3B panels, respectively) were examined for GFP fluorescence and Ci^155^ expression by immunofluorescence. Use of an antibody raised against Ci’s C-terminus detected only the active full length Ci^155^, but not the C-terminally truncated Ci^75^ transcriptional repressor form[32]. Please note that all images were acquired using fixed illumination/acquisition parameters and Ci^155^ expression reflected through application of a “Union Jack” lookup table to indicate regions of low (blue), medium (white) and high (red) levels of expression.

Ci^155^ expression in the *FRT82B* control discs showed a characteristic pattern of expression fof high levels in a dorsal-ventral stripe (DVS) that divided the disc into anterior and posterior compartments (Fig 3B, arrow). A small region of high Ci^155^ staining was also apparent at the posterior/dorsal edge (Fig 3B, arrowhead)[33], while intense signals at the ventral edge coincided with the disc folding over upon itself (Fig 3B asterisk). Please note that Ci^155^ DVS expression marks the front of the morphogenetic furrow (MF)[34], a morphological feature that, through the action of Hedgehog signalling, progresses in a posterior to anterior direction. Thereby acting to constantly redefine the regions of the EA discs’ anterior and posterior domains[3, 35].

In *hyd* ^*K7*.*19*^ EA discs (Fig 3C and 3D) the general Ci^155^ DVS staining pattern was perturbed (Fig 3D), exhibiting irregularities in its positioning, width and staining intensity. The presumed DVS (Fig 3D arrow) frequently undulated along the dorsal-ventral axis and exhibited significant broadening, as well as “arcs” of increased Ci^155^ staining spreading out into the posterior compartment (Fig 3D dashed region). Quantification of the area of medium-to-high Ci^155^ intensity staining (white+red) revealed >3-fold increase in *hyd* ^*k7*.*19*^ EA discs over control (Fig 3E).

**Fig 4:**
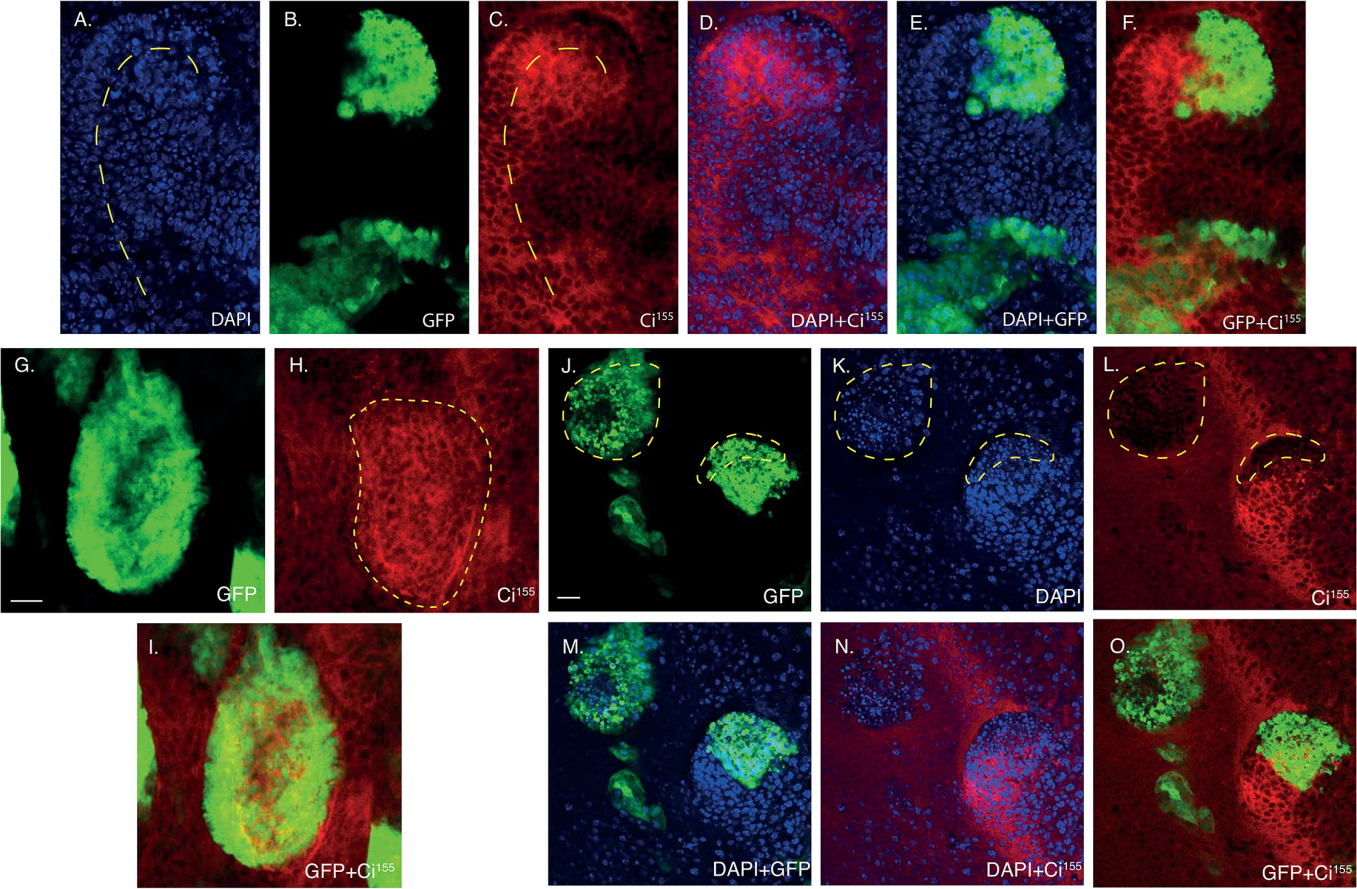
*hyd* ^*k7*.*19*^ clones exhibit distinct patterns of Ci^155^ expression. Confocal image sections of *hyd* ^*k7*.*19*^ EA discs imaged for direct GFP fluorescence (B,E,F,G,I,J,M,O) Ci^155^ immunofluorescence (C,D,F,H,I,L,N,O) and DAPI (A,D,E,K,M,N). (A-F) *hyd* ^*k7*.*19*^ discs exhibited curved arrays of nuclei (A, dashed line) that were reflected in the Ci^155^ DVS (C, dashed line). (G-I) Posterior *hyd* ^*k7*.*19*^ clones near the DVS exhibited increased Ci^155^ expression (H, dashed outline). (J-O) Anterior *hyd* ^*k7*.*19*^ clones near the DVS exhibited decreased Ci^155^ expression (L, low Ci^155^ marked by dashed lines, which are overlaid onto J,K). Scale bars = 10μm.

Closer examination of the DVS region of control EA discs revealed well-ordered nuclei showing a characteristic pattern of densely packed nuclei flanking a region of less-dense nuclei (Fig 3F, demarcated by dashed lines). The intense strip of Ci^155^ expression two-three cells wide (Fig 3H, arrow) marks the anterior boundary between the high and low nuclei densities (Fig 3F). Posterior to the Ci^155^ DVS, less intense Ci^155^ staining exhibited a characteristic ‘lattice-like’ staining pattern (Fig 3H) associated with differentiating photoreceptors. In contrast to the regulator patterns seen in the control, *hyd* ^*k7*.*19*^ EA discs exhibited two Ci^155^ DVS-like signals (Fig 3N, arrows). Potentially indicating the existence of two MFs within the one EA disc—a known effect associated with ectopic *hh* expression[36]. The DVS region also exhibited disorganised cell nuclei that formed ‘swirls’ (Fig 4A dashed line) that overlapped with Ci^155^ DVS staining (Fig 4C). Potentially indicating that Ci^155^-associated MF progression was disrupted.

Closer assessment of the effects on Ci^155^ expression within *hyd* ^*k7*.*19*^ clones revealed increased expression within clones located posterior to, and in the vicinity of, the DVS (Fig 4G–4I). Whereas infrequent *hyd* ^*k7*.*19*^ clones well within the anterior compartment exhibited reduced Ci^155^ expression (Fig 4J–4O, dashed lines). Therefore, in a spatially dependent manner, clonal loss of Hyd function resulted in both cell autonomous increases and decreases in Ci^155^ expression. Nevertheless, the predominant effect observed in *hyd* ^*k7*.*19*^ EA discs was increased Ci^155^ expression within and around the DVS, both in and outside of *hyd* ^*k7*.*19*^ clones.

**Fig 5:**
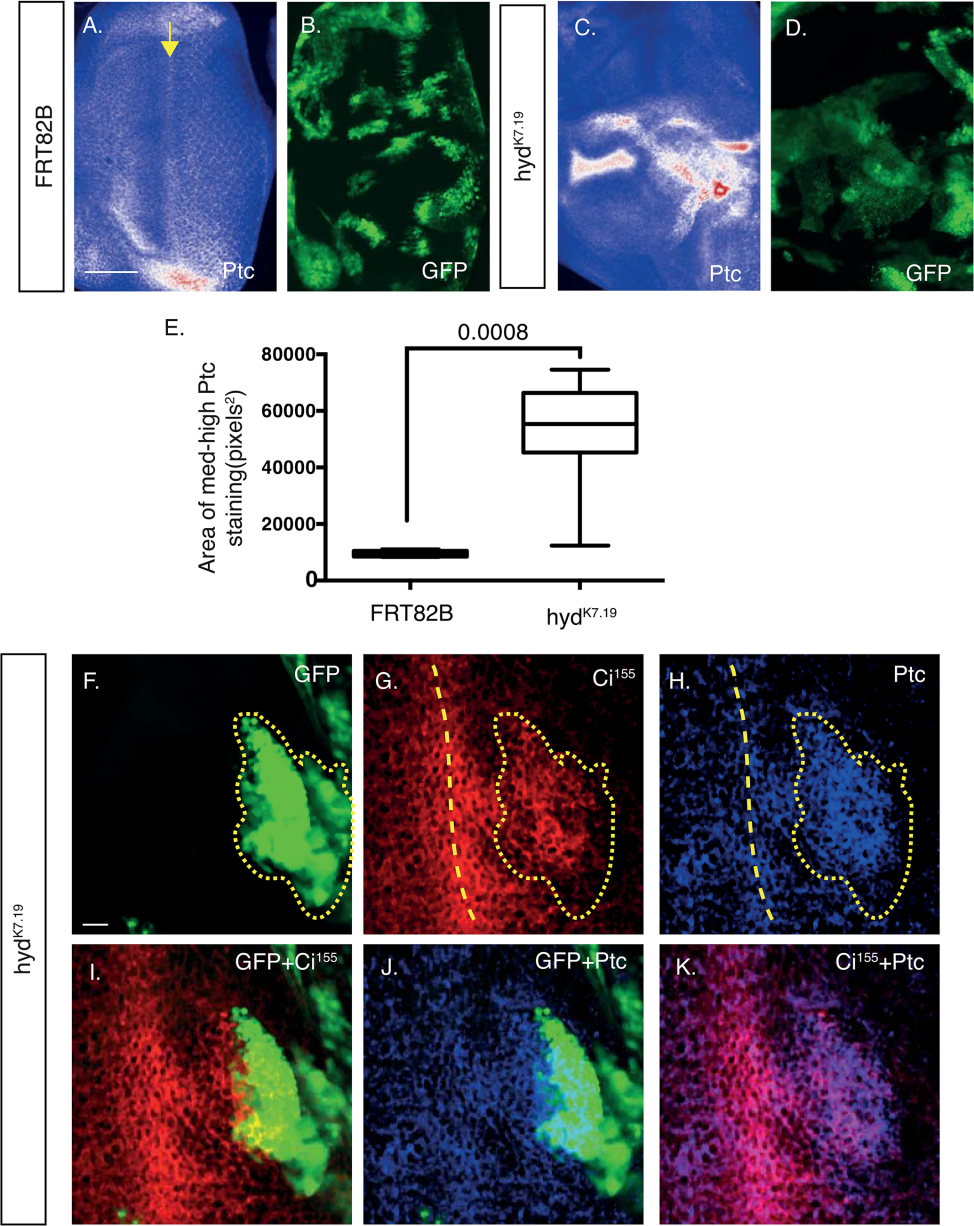
*hyd* ^*k7*.*19*^ EA discs exhibit abnormal Ptc expression. (A-D) Deconvolved widefield and (F-K) confocal image sections of control FRT82B (A,B) and *hyd* ^*k7*.*19*^ (C,D and F-K) EA discs imaged for GFP fluorescence and the indicated antigens for IF. “Union Jack” lookup table applied to Ptc images (A,C) to visualise low (blue), medium (white) and high (red) intensity levels. (E) Quantification of the area of medium and high Ptc signal. n = 3, s.e.m and indicated p value determined by Student’s t-test. (F-K) Overlapping expression of Ci^155^ and Ptc immunofluorescence within a *hyd* ^*k7*.*19*^ GFP-positive clone anterior to the Ci^155^ DVS (F yellow dotted outline, which is overlaid onto G,H). The yellow dashed line indicates the Ci^155^ DVS, which is overlaid onto H). Scale bars = 50μm (A-D) and 10μm (F-K).

**Fig 6:**
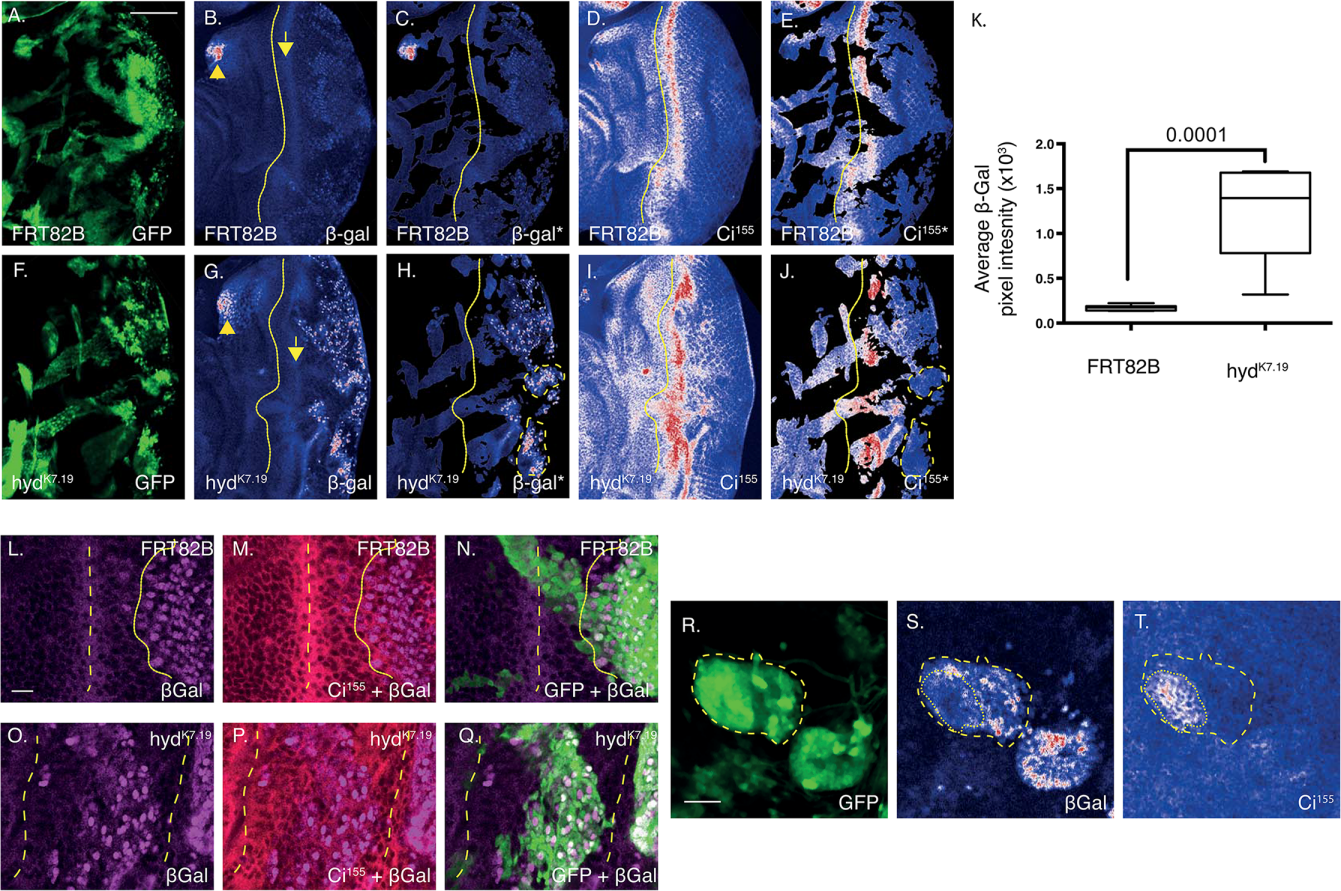
*hyd* ^*k7*.*19*^ EA discs exhibit increased *hh-lacZ*-associated β-Gal expression within the posterior compartment and DVS-region. Confocal image sections of *FRT82B* control (A-E, L-N)) and *hyd* ^*k7*.*19*^ (F-J, O-T) EA discs imaged for direct GFP fluorescence (A,F,N,Q,R), β-Gal (B,C,G,H,L-Q,S) and Ci^155^ immunofluorescence (D,E,I,J,M,P,T). (A-K) *hyd* ^*k7*.*19*^ EA discs exhibited increased β-Gal expression (H) relative to *FRT82B* controls (C). Non-clonal regions (GFP—ve regions) were ‘masked off’ to help visualise β-Gal and Ci^155^ expression only within GFP-positive clones (C,H and E,J, respectively). Yellow dotted lines indicate the division between anterior and posterior compartment (B—E and G-J). Dashed yellow lines indicate regions of high *hh* expression (H) and corresponding low Ci^155^ expression (E). (K) Quantification of the β-Gal average pixel intensity of the masked off images. n = 5, s.e.m and indicated p value determined by Student’s t-test. Scale bars = 50μm. (L-Q) *hyd* ^*k7*.*19*^ DVS regions exhibited abnormal β-Gal (O) and Ci^155^ (P) expression. Dashed lines indicate high Ci^155^ DVS expression (M,P), which are overlaid onto the other panels. The dotted line marks the anterior front of high β-Gal expression (L), which is overlaid on (M,N). (R-T) Two GFP positive *hyd* ^*k7*.*19*^ clones (R), located in the posterior compartment clearly overexpressed β-Gal (S). Of those clones, only one (R yellow dashed line, which is overlaid onto S,T) also harboured increased Ci^155^ expression (T). A specific clonal subregion (T, dotted line) with the clone coincided with low β-Gal expression (S, dotted line). Scale bars = 10μm.

**Fig 7:**
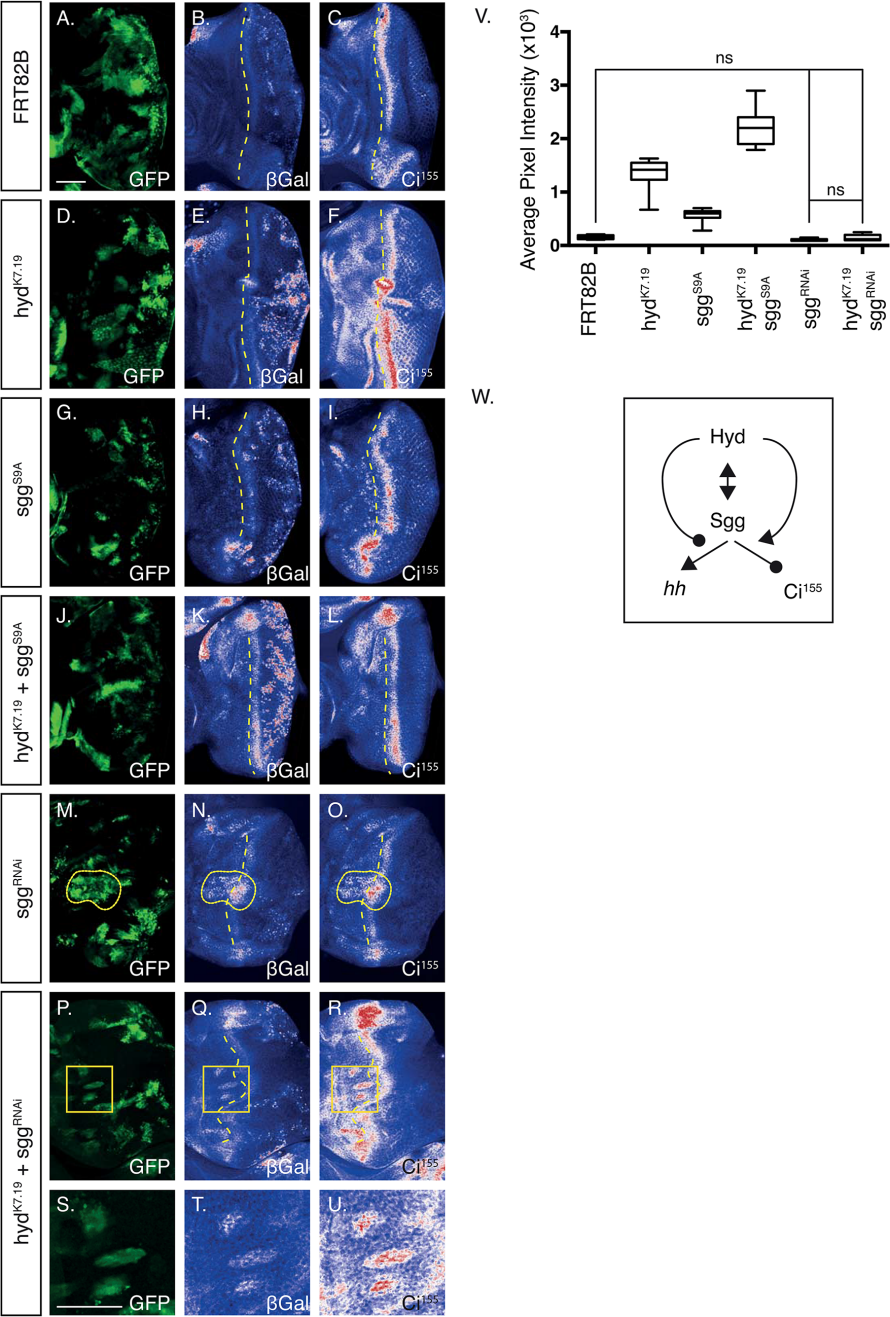
Sgg regulates *hh*-*lacZ* expression in both the posterior and anterior compartments. (A-U) Confocal images of EA disc of the indicated genotypes imaged for GFP (A,D,G,J,M,P,S) fluorescence and β-Gal (B,E,H,K,N,Q,T) and Ci^155^ (C,F,I,L,O,R,U) immunofluorescence. Dashed lines indicate the division between the anterior and posterior compartments, dotted lines indicate regions of high β-Gal and Ci^155^ expression within and anterior to the DVS region (N,O, respectively). The boxed regions (P-R) indicate a region harbouring three clones overexpressing β-Gal and Ci^155^ in the anterior compartment, which are enlarged in (S-U). (V) Boxplots of quantification of the average β-Gal pixel intensity of non-GFP masked off images (not shown). n = >5 for each genotype, s.e.m indicated. Statistical analysis by one-way ANOVA and Tukey’s multiple comparison tests, which revealed all comparisons to be statistically significant, except those indicated as non-significant (ns). (W) Potential model to explain the effects observed in the posterior EA disc. The double-headed arrow indicates a physical interaction, the single-headed arrow a positive regulatory action and the round-headed arrow a negative regulatory action. Scale bar = 50μm.

To establish if the increased Ci^155^ expression patterns translated into increased HhP activity, we next examined the protein product of one of Ci^155^ target genes, *ptc*. In control discs (Fig 5A and 5B), the posterior compartment expressed Ptc in a regular lattice-like pattern with a weak dorsal-ventral signal, reminiscent of the Ci^155^ DVS (Fig 5A, arrow). As with Ci^155^, *hyd* ^*K7*.*19*^ EA discs exhibited ectopic Ptc staining (white/red signals) that showed no clear, or exclusive, co-localisation with *hyd* ^*k7*.*19*^ GFP clones (compare Fig 5C and 5D). Quantification of the average Ptc signal intensity revealed a marked increase in the Ptc expression levels across the *hyd* ^*k7*.*19*^ EA disc (Fig 5E). Next we used co-immunofluorescence to directly assess whether particular clones located across the EA disc co-localised with altered Ci^155^ and Ptc expression (Fig 5F–5K). Only *hyd* ^*K7*.*19*^ clones located adjacent and posterior to the DVS demonstrated a clear positive correlation between ectopic Ci^155^ and Ptc expression (Fig 5G and 5H, respectively). However, when considering the pattern across the rest of the EA disc, neither ectopic expression of Ptc nor Ci^155^ exclusively co-localised within *hyd* ^*k7*.*19*^ clones (also see Figs 6 and 7). We therefore conclude that the presence of *hyd* ^*k7*.*19*^ clones within an EA disc elicits a generalised increase in HhP activity both within and outside of the clones. Importantly, aberrant Ci^155^ and Ptc expression patterns in *hyd* ^*k7*.*19*^ EA discs were effectively rescued by co-expression of the *UAS-hyd* ^*WT*^ transgene (S1 Fig). This effective rescue supported the idea that the aberrant Ci^155^ expression pattern was specifically caused by the loss of *hyd* function, rather than any other mutations carried on the *hyd* ^*k7*.*19*^-bearing chromosome arm.

### *hyd* ^*K7*.*19*^ EA discs exhibit increased *hh* expression

EA discs bearing *hyd* ^*k7*.*19*^ clones clearly exhibited abnormal Ci^155^ DVS patterns that suggested improper MF initiation/progression/termination. Due to Hh’s important role in both regulating Ci^155^ expression and MF initiation and progression, we sought to examine if *hh* mRNA expression was also abnormal. The altered effects of Ci^155^ expression outside of *hyd* ^*k7*.*19*^ clones also suggested that an extracellular signalling molecules derived from *hyd* ^*k7*.*19*^ cells could account for cell-non-autonomous effects. Previous work by Lee et al suggested that *hyd* ^*K7*.*19*^ mutant clones spatially misexpressed *hh* mRNA in the posterior compartment*[24].* Such spatial Hh misexpression, and subsequent paracrine-mediated activation of the HhP, could have accounted for the observed ectopic Ci^155^ expression outside of *hyd* ^*k7*.*19*^ clones. To support this hypothesis, we first wanted to confirm that *hyd* ^*K7*.*19*^ mutant cells spatially, and/or quantitatively, misexpressed *hh* mRNA. Using a *hh* lacZ enhancer trap (*hhP30*)[34] we were able to indirectly assess endogenous *hh* mRNA expression by determining β-galactosidase (β-Gal) activity and expression levels.

Previous work in the wing disc revealed that Ci^75^ repressed *hh* expression[37]. We therefore wished to see if expression of unprocessed Ci^155^ correlated with increased *hh* expression. To address this we used IF to examine co-localisation of *hh-lacZ-*derived β-Gal with Ci^155^ and the GFP signals marking *hyd* ^*k7*.*19*^ clones (Fig 6). To aid quantification, β-Gal and Ci^155^ images were acquired by fixed acquisition parameters and application of Image J’s “Union Jack” look-up-table, segmenting signal intensities into low (blue), medium (white) and high (red). Analysis of *FRT82B* control EA discs revealed (i) a thin, low-level dorsal-ventral ‘stripe’ of β-Gal expression in the middle of the posterior domain (Fig 6B, dotted line), (ii) high expression in the anterior-dorsal region (Fig 6B arrowhead) and (iii) low-level expression within the posterior compartment. Please note that non-recombined cells remained heterozygous for *hh-lacZ* and were therefore capable of expressing β-Gal.

Similar to control discs, *hyd* ^*k7*.*19*^ EA discs exhibited the same low-level dorsal-ventral ‘stripe’ expression (Fig 6G arrow), but had dramatically increased β-Gal expression within the posterior compartment (compare Fig 6B with 6G). To exclusively analyse the regions corresponding to GFP-positive mitotic clones we used thresholding of the GFP channel to mask out the β-Gal and Ci^155^ signals of non-GFP expression regions (Fig 6C, 6H, 6E and 6J, respectively). Comparing the masked *hyd* ^*K7*.*19*^ images revealed no clear co-localisation of increased Ci^155^ and β-Gal expression; e.g., two GFP-positive regions exhibiting high *hh* expression, but low Ci^155^ expression are indicated (Fig 6H and 6J, dashed lines). Quantification revealed *hyd* ^*k7*.*19*^ EA discs to express β-Gal >10-fold over that of *FRT82B* control (Fig 6K). In summary, Hyd potently suppressed *hh* expression in the posterior half of the posterior compartment of EA discs.

Closer examination of the MF region in control EA discs (Fig 6L–6N) revealed the expected increase in β-Gal expression (Fig 6L) 3–5 cell diameters posterior to the Ci^155^ DVS (Fig 6M)[3]. In contrast, *hyd* ^*k7*.*19*^ EA clones within the presumed MF region (Fig 6O and 6P) were associated with disordered β-Gal expression patterns (Fig 6O) flanked by regions of high DVS-like Ci^155^ expression (Fig 6P, dashed lines). *hyd* ^*k7*.*19*^ EA discs also exhibited rare posterior *hyd* ^*K7*.*19*^ clones that overexpressed both β-Gal and Ci^155^ (Fig 6R–6T, dashed line). Yet, even within those clones there appeared to be some degree of mutual exclusivity in Ci^155^ and β-Gal/*hh* expression (compare the dotted region within the marked clone in Fig 6S and 6T). In summary, due to the general negative correlation between Ci^155^ and *hh* expression, our findings do not support a role for Ci^75^-mediated suppression of *hh* in the EA disc. A finding that is in agreement with the genetic approach taken by Lee et al[24].

### *sgg* ^*RNAi*^ rescues *hyd* ^*k7*.*19*^-associated ectopic *hh* expression

The marked increase in *hh* gene expression in *hyd* ^*k7*.*19*^ EA discs potentially explained some of the EA disc-wide effects on Ci^155^ and Ptc expression. However, what remained unclear was how *hh* expression was being misregulated within *hyd* ^*k7*.*19*^ clones? Although Ci can regulate *hh* ligand expression in the wing disc[37], we found no positive correlation between increased Ci^155^ expression and *hh* overexpression in *hyd* ^*k7*.*19*^ clones (Fig 6H, 6J, 6S and 6T). We next turned our attention to Hyd’s binding partner, Sgg, a kinase implicated in regulating the transcriptional output of diverse signalling pathways[4]. To directly address a role for Sgg in the *hyd* ^*k7*.*19*^–associated overexpression of *hh* we chose to increase or decrease Sgg function in *hyd* ^*K7*.*19*^ clones. Use of *UAS*-driven transgenes allowed us to express either an active *sgg* mutant (*sgg* ^*S9A*^) that is refractory to insulin-signalling mediated inhibition[38], or *sgg* ^*RNAi*^ specifically within *FRT82B* control or *hyd* ^*k7*.*19*^ clones (Fig 7).

Clonal overexpression of Sgg^S9A^ alone had no dramatic effect on Ci^155^ expression patterns either within GFP clones or on the EA disc as a whole (compare Fig 7A and 7C with 7G and 7I). However, there was an apparent increase in β-Gal expression within the posterior compartment (compare Fig 7B with 7H). These observations suggested that Sgg^S9A^ promoted *hh* expression in the posterior domain without significantly affecting Ci^155^ expression. Overexpressing Sgg^S9A^ within *hyd* ^*K7*.*19*^ clones reduced Ci^155^ expression to that of control (compare Fig 7C, 7F and 7L) and promoted β-Gal expression within the dorsal-ventral stripe region (compare Fig 7B, 7E and 7K). Therefore, our data indicated that in a *hyd* ^*k7*.*19*^ background Sgg^S9A^ overexpression suppressed ectopic Ci^155^ expression and promoted *hh* expression.

In contrast to *sgg* ^*S9A*^, *sgg* ^RNAi^ alone had no obvious effects on β-Gal or Ci^155^ expression in the posterior, but did increase their expression in regions within, and anterior to, the DVS region (compare Fig 7B, 7N and 7C and 7O, dotted lines, respectively). In a *hyd* ^*k7*.*19*^ background *sgg-RNAi* reduced Ci^155^ staining in the posterior, but increased its expression levels within, and anterior to, the DVS (compare Fig 7F and 7R). A very similar pattern was also observed for β-Gal (compare Fig 7E and 7Q). Together these observations indicated that within the posterior compartment, Sgg functions to suppress Ci^155^ and promote *hh* expression. Interestingly, rounded anterior clones within *hyd* ^*k7*.*19*^+*sgg* ^*RNAi*^ EA discs exhibited elevated Ci^155^ staining that co-localised with increased *hh* expression (Fig 7P–7R and 7S–7U). These observations suggested that within the anterior compartment, Sgg functions to repress both Ci^155^ and *hh* expression.

The masking technique described in Fig 6 allowed quantification of clonal β-Gal average intensities within the posterior compartment. Analysis revealed Sgg^S9A^ overexpression caused a two- and >20-fold increase in β-Gal expression in comparison to *hyd* ^*k7*.*19*^ or *FRT82B* control discs, respectively (Fig 7V). The increased β-Gal expression in *hyd* ^*k7*.*19*^ + *sgg* ^*S9A*^ EA discs indicated potential co-operation between loss of Hyd and gain of Sgg function in promoting *hh*/β-Gal expression. In agreement with the IF images, *sgg* ^RNAi^ in a *hyd* ^*k7*.*19*^ background reduced *hh*-β-Gal expression levels back to that of *FRT82B* control levels. In summary, our image and quantification data indicated that Sgg regulated *hh* expression in both the posterior and anterior compartments and modified *hyd* ^*k7*.*19*^-associated ectopic Ci^155^ and *hh* expression patterns.

Taken together our data suggested that, within the central and posterior regions, loss of Hyd function cell autonomously (i) promoted Sgg-mediated promotion of *hh* expression—that potentially accounted for the increased Ci^155^ expression outside of *hyd* ^*k7*.*19*^ clones and (ii) inhibited Sgg-mediated repression of Ci^155^ expression—that potentially contributed to the increased Ci^155^ expression within *hyd* ^*k7*.*19*^ clones. Please note that our observations cannot exclude a role for autocrine/paracrine Hh-mediated increases in Ci^155^ expression within *hyd* ^*k7*.*19*^ clones. A model of the physical and functional relationships between Hyd and Sgg is depicted in Fig 7W.

### *sgg* ^*RNAi*^ rescues the *hyd* ^*K7*.*19*^ adult eye phenotype

**Fig 8:**
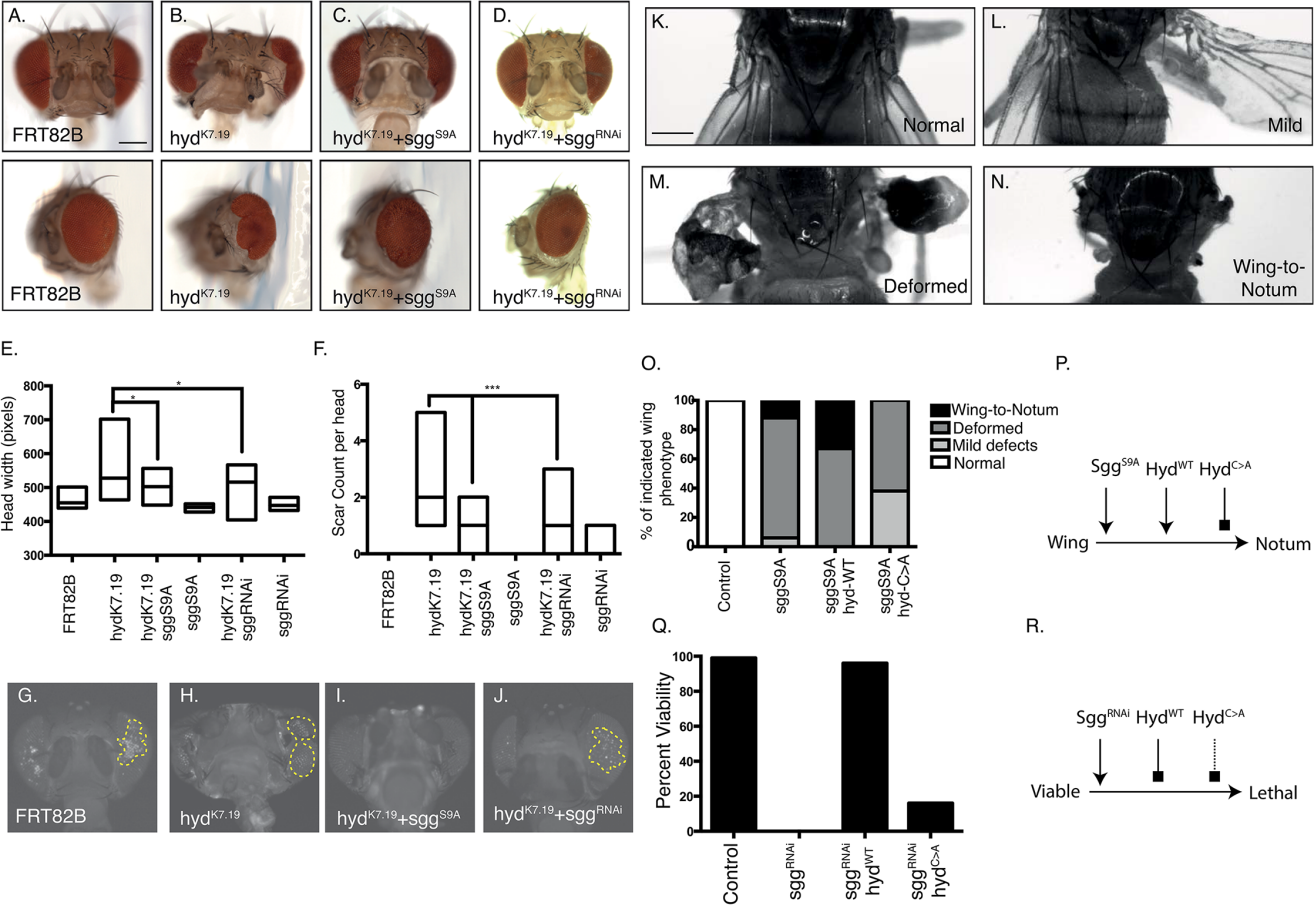
Sgg and Hyd genetically interact to govern animal viability and head and wing development. (A-J) Sgg perturbation modifies the *hyd* ^*k7*.*19*^ head phenotype. (A-D) Brightfield images of adult *Drosophila* heads of the indicated genotypes shown either ‘head on’ (upper panels) or ‘side on’ (lower panels). Both gain (C) and loss (D) of *sgg* function appeared to rescue the *hyd* ^*k7*.*19*^ phenotype. Boxplots indicating head width (E, n = ≥8 for each genotype) and counts of eye scars (F, n = ≥8 for each genotype) of the indicated genotypes, with statistical analysis by one-way ANOVA (E) and Fishers exact test (F) revealed statistical significance (asterisks). (G-J) Representative GFP fluorescent signals in adult *Drosophila* heads of the indicated genotypes revealed only *hyd* ^*k7*.*19*^ *+sgg* ^*S9A*^ animals lack a GFP signal (n = ≥4 for each genotype). Scale bars = 175μm. (K-P) *hyd* ^*WT*^ overexpression promotes the *sgg* ^*S9A*^-mediated wing phenotype. (K-N) Brightfield images of adult *Drosophila* wings showing (K) normal, (L) mildly deformed, (M) severely deformed and (N) wing-to-notum phenotypes. (O) Percentage of adult wing phenotypes of *vg-GAL4* flies expressing the indicated transgenes, revealing that the *hyd* ^*WT*^ transgene enhanced, and the *hyd* ^*C>A*^ transgene suppressed the severity of the *sgg* ^*S9A*^ wing defects (n ≥12 for each genotype). (P) Model showing the genetic interaction between *sgg* ^*S9A*^ and *hyd* UAS-transgenes with respect to the wing-to-notum phenotype. Arrows indicate promotion and blockhead arrows inhibition. (Q-R) *hyd* ^*WT*^ overexpression rescues *sgg* ^*RNAi*^-mediated embryonic lethality. Percentage viability of *sca-GAL4* flies expressing the indicated transgenes revealed a >95% rescue of embryonic lethality upon co-expression with the *UAS-hyd* ^*WT*^, but not *UAS*-*hyd* ^*C>A*^, transgene (16 individual crosses per genotype). (R) Model showing the genetic interaction between *sgg* and *hyd* UAS-transgenes. Arrows indicate promotion, blockhead arrows inhibition and dotted blockhead arrow weak inhibition. Scale bar = 250μm.

Next we wished to determine if modulation of Sgg activity modified the adult *hyd* ^*K7*.*19*^ head phenotype (Fig 8A–8D). Surprisingly both *sgg* ^*S9A*^ and *sgg* ^*RNAi*^ rescued the *hyd* ^*K7*.*19*^ phenotype (compare Fig 8B with 8C and 8D, respectively). Quantification of the phenotypic effects revealed a significant decrease upon perturbation of *sgg* function (Fig 8E and 8F, respectively), with *sgg* ^*S9A*^ resulting in the more robust rescue. To ensure that mutant clones were persisting and contributing to adult structures we examined the adults’ heads for GFP signals (Fig 8G–8J). An absence of GFP positive clones in the *hyd* ^*k7*.*19*^+*sgg* ^*S9A*^ adult heads (Fig 8I) suggested that the combined loss of *hyd* and gain of *sgg* function (*hyd* ^*k7*.*19*^ + *sgg* ^*S9A*^) eliminated the mutant cells from the developing EA disc/head. Hence, it appears that *sgg* loss of function (*hyd* ^*k7*.*19*^ *+sgg* ^*RNAi*^), rather than eliminating cells, actively rescued the signalling defects associated with the *hyd* ^*k7*.*19*^ phenotype. Comparing the molecular phenotypes of the Sgg^S9A^ and *sgg* ^*RNAi*^ rescue EA discs (Fig 7J–7L and 7P–7U, respectively) revealed that the reduction in *hh* expression correlated with the ‘true’ phenotypic rescue by *sgg* ^*RNAi*^.

### *hyd* and *sgg* genetically interact to regulate animal viability and wing development

Our work in the eye disc indicated that *hyd* and *sgg* exhibited a complex genetic interaction to influence EA disc development. We next used *UAS-GAL4*-based overexpression and RNAi studies to confirm that *hyd* and *sgg* genetically interacted in other imaginal discs/organs. The wing disc was chosen based upon *hyd* ^*K3*.*5*^ clones phenocopying the Ci^155^ and *hh* effects observed in the EA disc[24] and importance Hh signalling in its development[39]. Sgg^S9A^ overexpression in the developing wing disc by the *vestigal*-*GAL4 (vg-GAL4)* driver (Fig 8K–8P) resulted in deformed wings (Fig 8L and 8M) and with less frequency wing-to-notum transformation(Fig 8N)[38]. Co-expression of *UAS-hyd* ^*WT*^ enhanced the Sgg^S9A^ phenotype resulting in a significant increase in the percentage of flies exhibiting wing-to-notum transformation, whereas E3 defective *UAS-hyd* ^*C>A*^ suppressed the Sgg^S9A^ phenotype (Fig 8O). These observations suggested that Sgg and Hyd^WT^, but not Hyd^C>A^, co-operated in promoting wing-to-notum transformation (Fig 8P). A similar positive relationship was also observed when using *scaborous*-GAL4 (*sca-GAL4*) to drive *sgg-RNAi* in a number of organs that includes the wing disc[40]. Remarkably, the embryonic lethality associated with *sgg-RNAi* was effectively rescued by co-expression of *UAS-hyd* ^*WT*^, but not *UAS-hyd* ^*C>A*^ (Fig 8Q). These observations further support a strong genetic interaction between *hyd* and *sgg* in diverse aspects of *Drosophila* development. Additionally, it appeared that Hyd^WT^, but not Hyd^C>A^, efficiently functioned to potentially promote Sgg function (Fig 8R). Please note that our *vg*- and *sca*-GAL4 studies suggested that Hyd promoted Sgg function, yet in the EA Hyd appeared to suppress Sgg function. Potential explanations of this apparent contradiction are detailed in the discussion below.

## Conclusion

In conclusion, our results identified a genetic and physical interaction between *hyd*/Hyd and *sgg*/Sgg, as well as a role in regulating imaginal disc development, embryonic viability and *hh* and Ci^155^ expression.

## Discussion

### Sgg and Hyd regulate *hh* expression

Both Hyd and Sgg regulate *hh* expression in the posterior domain, with Sgg promoting and Hyd suppressing *hh* expression. Our epistasis experiments in the EA disc with the *sgg* ^*S9A*^ and *sgg* ^*RNAi*^ transgenes also revealed that *sgg* functions either downstream of, or parallel to, *hyd* in regulating *hh* expression. Therefore within the posterior EA disc we suggest that Hyd normally represses Sgg’s ability to promote *hh* expression. Combined with the fact that the two proteins physically interact, we believe that they function in the same signalling pathway, albeit with opposing effects on *hh* expression.

The observed general increase in Ci^155^ expression within *hyd* ^*k7*.*19*^ discs located near the DVS provided a potential mechanism of regulating *hh* expression[37]. While we did not directly address Ci^75^ expression levels, we may infer that increased Ci^155^ expression may have resulted from decreased Ci^75^ generation and a subsequent de-repression of *hh* expression. Our observation that *hyd* ^*k7*.*19*^ clones located well within the posterior compartment exhibited increased *hh*, but low Ci^155^ expression, failed to support a role Ci^75^-mediated regulation of *hh* expression. Furthermore, Sgg^S9A^ overexpression, which would be predicted to promote Ci^75^ production, also promoted *hh* expression.

Sgg^S9A^’s ability to promote ectopic *hh* expression raised the possibility that transcriptional control via other signalling pathway could also be involved. GSK3β governs the activity of multiple transcription factors[4, 41] that could potentially influence *hh* transcription. Of *hh’s* known transcriptional (Engrailed[42], Master of Thickveins[43], Serpent[44]) and epigenetic regulators (PRC1/2 and Trithorax[45] and Kismet[46]) none are reported to bind to Hyd or Sgg (Biogrid/INTact databases). Hence there is no clear candidate to potentially explain how Sgg/GSK3β’s might regulate *hh* expression.

### Hyd Suppresses Ci^155^ expression

Within *hyd* ^*k7*.*19*^ clone-bearing EA discs, elevated Hh-mediated paracrine signalling most likely accounted for the increased Ci^155^ expression outside of *hyd* ^*k7*.*19*^ clones. Whereas within *hyd* ^*k7*.*19*^ clones themselves, Hyd can cell autonomously influence Ci^155^ expression levels independent of its effects on *hh* transcription[24]. We clearly observed marked changes in Ci^155^ expression within *hyd* ^*k7*.*19*^ clones relative to surrounding control cells, which are presumably exposed to similar local Hh expression levels. Therefore, cell-intrinsic genetic differences between cells, rather than distinct Hh levels, potentially explained the Ci^155^ expression patterns. We hypothesise that cell autonomous effects on Ci^155^ expression observed within *hyd* ^*k7*.*19*^ clones may be due to reduced Sgg-mediated Ci^155^ proteolysis. In summary, we believe that Ci^155^ expression levels across the *hyd* ^*k7*.*19*^ EA disc are governed by both Hh-ligand-dependent and-independent mechanism that may both rely on Hyd and Sgg function.

Ci^155^ expression is post-translationally controlled by two distinct Cullin-based E3 complexes that are distinguished by their substrate-specificity factors Slmb[7] and Rdx[8, 9] and their spatially restricted actions. In general, the Cul-1^Slmb^ complex promotes Ci^155^ processing in the anterior compartment, whereas the Cul-3^Rdx^ complex promotes Ci^155^ degradation in the posterior. However, Cul-1 and-3 activity overlap around the MF[47], which raises the possibility of both Cul-1^Slmb^-mediated Ci^155^ processing and Cul-3^Rdx^-mediated Ci^155^ degradation[47, 48] occurring within the same cell. Due to the *hyd* ^*k7*.*19*^ EA discs’ abnormal Ci^155^ DVS patterns (i.e., broader, irregular, posterior extensions) it was possible that Cul-associated activities were also spatially abnormal around an irregular morphogenetic furrow. Hence, we hypothesise that misexpression of Cul-associated E3 activities may underlie the numerous Ci^155^ expression defects observed within *hyd* ^*k7*.*19*^ clones. EDD’s ability to bind Cul-3[49] also supports a potential role for Hyd in influencing Cul-3^Rdx^-mediated Ci^155^ ubiquitylation and degradation.

### Hyd regulates imaginal disc development

*hyd* ^*k7*.*19*^ EA discs exhibiting ectopic *hh* expression would lead to abnormal paracrine Hh signalling and irregular MF progression. Disruption of such an important morphological landmark as the MF may have altered the discs’ anterior-posterior, dorsal-ventral and lateral-medial axes to disrupt spatial information and consequentially alter cell fates (e.g., eye to head capsule). Our studies in different imaginal discs and tissues clearly identify a strong genetic interaction between *hyd* and *sgg* in controlling *Drosophila* development. Due to the essential roles for Hh signalling in development, we hypothesise that defects in HhP activity underlies a significant component of the observed mutant phenotypes.

Within the EA disc, Wingless (Wg) and Hh morphogen signalling antagonise each other’s actions to specify the EA discs’ cellular fate and promote development of distinct adult head structures[50]. As in the EA disc, both morphogen signalling pathways also play essential role in wing disc development[3, 51]. Due to Sgg’s key roles in both morphigen signalling pathways perturbed Wg signalling could also contribute to the *hyd* ^*k7*.*19*^ mutant phenotype. The previously reported partial rescue of the *hyd* ^*K3*.*5*^ adult head phenotype upon loss of *hh* function[24], clearly suggested the additional involvement of other Hh-ligand-independent effects. Within the HhP, Hyd’s potential ability to influence Sgg-mediated Ci^155^ expression could be one such Hh-ligand-independent component. However, an effect totally independent of the HhP, such as the Wg pathway, could also contribute to the *hyd* ^*K7*.*19*^ adult head phenotype.

While Hh plays an important role in wing development, abnormal Wg signalling plays a known role in the wing-to-notum transformation[38]. EDD’s ability to affect β-catenin activity[30, 52] supports a potential evolutionarily conserved role for Hyd in Wg pathway signalling. Therefore, future work should focus on simultaneously investigating both Wg- and Hh-mediated signalling in *hyd* mutant tissue. Although we are uncertain as to exact molecular mechanisms involved, our sequencing of the *hyd* ^*k7*.*19*^ allele, in combination with our *hyd* transgene experiments in the eye and wing discs, clearly support an important role for Hyd’s HECT-associated E3 activity in regulating Sgg function and controlling *Drosophila* development.

### Epistatic relationship between *hyd* and *sgg*

At the morphological level the epistatic relationships observed in the eye and wing disc appear contradictory. In the eye, Hyd appeared to repress Sgg function while in the wing disc it appeared to promote Sgg function. A simple explanation may reside in technical differences between generating cells totally lacking full-length Hyd (*hyd* ^*k7*.*19*^) versus those experiencing a reduction/overexpression. The difference in the two systems is clearly demonstrated by an absence of an adult head phenotype upon EA disc clonal overexpression of Sgg^S9A^ compared to the dramatic adult wing phenotypes upon *vg*-GAL4-mediated Sgg^S9A^ overexpression.

An alternative explanation resides in tissue-specific differences between eye and wing imaginal discs. This notion is supported by the striking fact that *hyd* hemizyogous mutant animals harbour hyperplastic wing and hypoplastic haltere discs[11]. Hence loss of Hyd function can produce diametrically opposed effect in different types of imaginal discs. Additionally, the functional relationship between Hyd and Sgg is not a simple one, whereby Hyd may (i) promote Sgg-mediated repression of Ci^155^ expression and yet (ii) inhibit Sgg-mediated promotion of *hh* expression (see Fig 7W). Taking the EA disc as a whole, Hyd can apparently both promote and inhibit distinct functional aspects of Sgg and Hedgehog signalling. Discrepancies at the morphological level may therefore potentially reflect Hyd’s differential ability to promote and inhibit distinct Sgg functions at the molecular level.

With tissue-specific requirements in mind, development of a particular tissue may be more susceptible to disruption of one of Hyd/Sgg’s Hh-associated functions than the other. For example, the genetic epistasis observed in the EA disc suggested that regulation of *hh* was the critical determinant for the disc’s correct development—highlighting Hyd’s importance in repressing Sgg-mediated *hh* expression (left arm of Fig 7W). In contrast, Hyd’s ability to promote Sgg-mediated inhibition of Ci^155^ (right arm of Fig 7W) may be the critical determinant for promoting abnormal wing development.

In summary our findings implicate both Sgg and Hyd as important regulators of *hh* ligand expression, HhP activity and imaginal disc development. Hyd may influence Sgg to utilise mechanistically independent actions to control initiation of (*hh* expression) and the response to (Ci^155^ expression) Hedgehog signalling. We hypothese that Hyd and Sgg act to establish distinct Hedgehog signalling cell states—e.g., (A) cells capable of producing Hh and but not responding to Hh stimulation and (B) cells only being able to respond to, but not produce, Hh. Such rigid cell states could help establish and subsequently enforce spatial divisions, whereas transitions between them could also allow morphogenetic elements like the MF to function as it moves across the EA disc.

Hyd’s ability to regulate Hh signalling provides it with the potential means to govern important cellular signalling pathway involved in both animal development and adult tissue homeostasis. These potential abilities may help to explain the dramatic phenotypes observed in homozygous *hyd* ^*K7*.*19*^ larvae[11], *Ubr5* null mice[53], conditionally mutant adult *Ubr5* mice (MD, manuscripts in preparation) and human cancers[54–56].

## Supporting Information

S1 Fig Expression of a *UAS-hyd* ^*WT*^ transgene rescues *hyd* ^*k7*.*19*^—associated aberrant Ci^155^ and Ptc expression patterns. Confocal images of *UAS-hyd* ^*WT*^; *FRT82B hyd* ^*k7*.*19*^ EA discs imaged, left to right, for direct GFP fluorescence, Ci^155^ and Ptc immunofluorescence and the indicated combinations. These discs exhibit relatively normal Ci^155^ and Ptc expression patterns, indicating an effective rescue of the *hyd* ^*k7*.*19*^ phenotype by overexpression of Hyd^WT^.
(PDF)
