# Supplementary figures and images for: Regulation of hedgehog Ligand Expression by the N-End Rule Ubiquitin-Protein Ligase Hyperplastic Discs and the Drosophila GSK3β Homologue, Shaggy
Source: PLoS One. 2015 Sep 3;10(9):e0136760. doi: 10.1371/journal.pone.0136760 (PMC4559392; doi:10.1371/journal.pone.0136760)

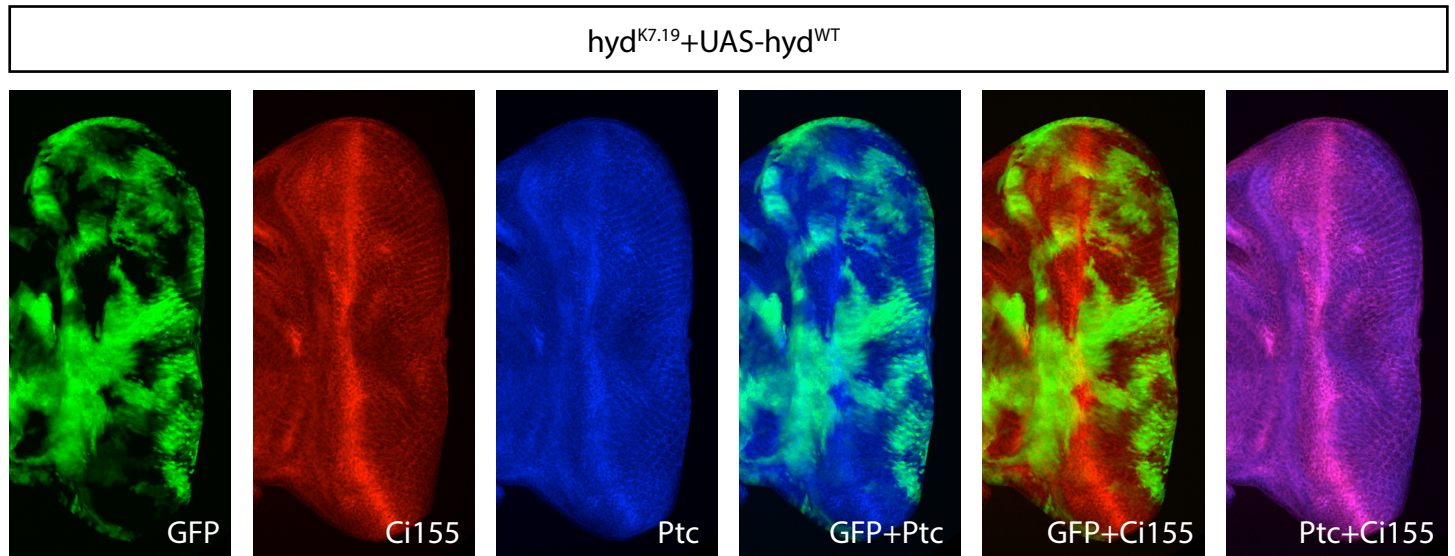

Supplement: S1 Fig — Confocal images of UAS-hyd WT; FRT82B hyd k7.19 EA discs imaged, left to right, for direct GFP fluorescence, Ci155 and Ptc immunofluorescence and the indicated combinations. These discs exhibit relatively normal Ci155 and Ptc expression patterns, indicating an effective rescue of the hyd k7.19 phenotype by overexpression of HydWT. (PDF) [file pone.0136760.s001.pdf]
